# GENDER DIFFERENCES IN THE EXPERIENCE OF AGE-RELATED MICROAGGRESSIONS

**DOI:** 10.1093/geroni/igae098.3254

**Published:** 2024-12-31

**Authors:** Hannah Lewis, Crystal Hecht, Jeff Buchanan

**Affiliations:** University of North Dakota, Grand Forks, North Dakota, United States; Minnesota State University Mankato, Mankato, Minnesota, United States; Minnesota State University Mankato, Mankato, Minnesota, United States

## Abstract

Microaggressions are subtle forms of discrimination which occur in interpersonal interactions. This construct has been examined individually in the context of sexism, and more recently, ageism. Despite society’s gendered view on aging, the intersection of how gender impacts the experience of age-related microaggressions is understudied. The current study utilized existing survey data from Lewis et al. (2023) to examine how intersectional identities of age and gender influence the experience of age-related microaggressions. The original study utilized an online survey to ask older adults (n=200, 57% females) about their experiences with 20 age-related microaggressions. Frequency analyses were used to identify gender differences by item, with a difference of >20% serving as a threshold for defining meaningful gender differences. Gender differences existed on items in all six variables measured (frequency, cause, relationship, location, emotional response, and behavioral reaction). Chi-squares tests of independence were also run to examine these differences on a larger scale. The results suggested gender had a significant relationship with frequency χ2(5)=6.64,p =.005, cause χ2(7)=157.61,p <.001, and emotional reaction χ2(2)=7.37, p =.03. Namely, women more often reported experiencing microaggressions, believed these interactions occurred because of their gender, and had negative emotional responses. Overall, the results suggest a difference in the experience and perception of these interactions. These results highlight a need for educational programming on ageism to incorporate a discussion on intersectional identities. Future studies should utilize focus groups to gain a better understanding of the role gender identity plays in the context of age-related microaggressions.

